# Geographical Variations in the Clinical Management of Colorectal Cancer in Australia: A Systematic Review

**DOI:** 10.3389/fonc.2018.00116

**Published:** 2018-05-16

**Authors:** Fiona Crawford-Williams, Sonja March, Michael J. Ireland, Arlen Rowe, Belinda Goodwin, Melissa K. Hyde, Suzanne K. Chambers, Joanne F. Aitken, Jeff Dunn

**Affiliations:** ^1^Institute for Resilient Regions, University of Southern Queensland, Springfield Central, QLD, Australia; ^2^School of Psychology and Counselling, University of Southern Queensland, Springfield Central, QLD, Australia; ^3^Cancer Research Centre, Cancer Council Queensland, Fortitude Valley, QLD, Australia; ^4^Menzies Health Institute Queensland, Griffith University, Southport, QLD, Australia; ^5^Prostate Cancer Foundation of Australia, St Leonards, NSW, Australia; ^6^Exercise Medicine Research Institute, Edith Cowan University, Perth, WA, Australia; ^7^School of Public Health and Social Work, Queensland University of Technology, Brisbane, QLD, Australia; ^8^School of Social Science, University of Queensland, Brisbane, QLD, Australia; ^9^School of Medicine, Griffith University, Brisbane, QLD, Australia

**Keywords:** colorectal cancer, rural health, health disparity, cancer treatment, systematic review

## Abstract

**Background:**

In Australia, cancer survival is significantly lower in non-metropolitan compared to metropolitan areas. Our objective was to evaluate the evidence on geographical variations in the clinical management and treatment of colorectal cancer (CRC).

**Methods:**

A systematic review of published and gray literature was conducted. Five databases (CINAHL, PubMed, Embase, ProQuest, and Informit) were searched for articles published in English from 1990 to 2018. Studies were included if they assessed differences in clinical management according to geographical location; focused on CRC patients; and were conducted in Australia. Included studies were critically appraised using a modified Newcastle–Ottawa Scale. PRISMA systematic review reporting methods were applied.

**Results:**

17 articles met inclusion criteria. All were of high (53%) or moderate (47%) quality. The evidence available may suggest that patients in non-metropolitan areas are more likely to experience delays in surgery and are less likely to receive chemotherapy for stage III colon cancer and adjuvant radiotherapy for rectal cancer.

**Conclusion:**

The present review found limited information on clinical management across geographic regions in Australia and the synthesis highlights significant issues both for data collection and reporting at the population level, and for future research in the area of geographic variation. Where geographical disparities exist, these may be due to a combination of patient and system factors reflective of location. It is recommended that population-level data regarding clinical management of CRC be routinely collected to better understand geographical variations and inform future guidelines and policy.

## Introduction

Australia and New Zealand have the highest incidence rates of colorectal cancer (CRC) in the world, and it is the second leading cause of cancer death in Australia (1, 2). Around 80 Australians die each week from CRC; however, if detected early it can be treated successfully (3). The Australian National Health and Medical Research Council and Cancer Australia have developed evidence-based guidelines for optimal care in the clinical management of CRC (4, 5). These guidelines recommend that patients should receive site- and stage-specific care including preoperative assessment, surgery, and adjuvant therapy where appropriate. Specifically, primary surgical resection is recommended for stage I to III CRC, except for low-grade stage I where local excision is appropriate; adjuvant chemotherapy for all node-positive colon cancers; adjuvant preoperative or postoperative radiotherapy for high risk rectal cancers; and chemotherapy for metastatic CRC. The guidelines also recommend less than 30 days between diagnosis and surgery, and receipt of treatment in specialist cancer centers or from specialist surgeons (5). To date, it is unclear whether these guidelines are adhered to uniformly across Australia.

In Australia, survival from CRC differs according to geographical location, with mortality rates higher in regional and remote areas compared to major cities (1, 6–8). Geographical variations in access to recommended treatments may contribute to the noted disparities in survival and other outcomes. Only 78 sites across Australia deliver radiotherapy treatment (42% private providers), with the majority of these located in capital cities or major regional centers (9). An analysis of available radiotherapy services in Australia in 2009 found that only 38% of cancer patients for whom radiotherapy was the appropriate treatment could be treated within the current service capacity, with lower percentages expected in regional and rural Australia (10). Geographical differences in surgery and chemotherapy also exist as there are reported gaps in the percentage of non-metropolitan hospitals with medical oncologists or specialist surgeons, and reports of administration of chemotherapy by staff without oncology training (11, 12).

Residents in metropolitan areas have increased access to services, and access to hospitals and surgeons with higher caseloads; factors known to be associated with better clinical outcomes (13, 14). However, few population-level datasets in Australia include comprehensive treatment data or clinical management information (15), and to date, there has been no aggregation and synthesis of available data. This evidence gap hampers our understanding of disparities in clinical care and how these might influence cancer outcomes. A preliminary survey of the literature regarding geographic disparities in outcomes for patients diagnosed with CRC highlighted a lack of clear, consistent findings and identified a need for a more in-depth examination of differences in clinical management (8). Thus, the primary aim of this systematic review was to understand the nature of geographical variations in the clinical management of CRC (including surgery, chemotherapy, and radiotherapy) in Australia, incorporating clinical reports as well as peer-reviewed literature.

## Methods

The review methodology was planned and carried out following the PRISMA statement for the conduct and reporting of systematic reviews (16). The review protocol was registered with PROSPERO; registration number CRD42016042666 (https://www.crd.york.ac.uk/PROSPERO/display_record.asp?ID=CRD42016042666).

### Eligibility Criteria

Studies were included if the data were from cohorts of Australian individuals with CRC; reflected outcomes pertaining to clinical management; and compared non-metropolitan vs metropolitan patients. Qualitative studies, review articles, editorials, books, commentaries, and conference abstracts were excluded.

### Search Strategy

PubMed, CINAHL, Embase, ProQuest, and Informit databases were searched for articles published in English from 1990 to 26th February 2018. Search strings included terms relating to “colorectal cancer” or “bowel cancer,” “clinical management,” “treatment,” “chemotherapy,” “radiotherapy,” or “surgery.” Terms relating to geographical disparities included “metropolitan,” “urban,” “rural,” “remote” or “regional,” and “Australia.”

Gray literature searches were conducted through targeted Internet searches of state and federal government health websites, non-government cancer association (e.g., state Cancer Council groups) websites, web search engines (Google), and manual hand searching of reference lists of included articles.

### Screening and Data Extraction

After removing duplicates, titles and abstracts were independently screened by two reviewers for relevance according to the selection criteria. Full text versions of potentially eligible articles were then assessed for inclusion by two independent reviewers. Reviewer discrepancies were discussed and resolved within the project team where necessary.

Criteria for data extraction were determined prior to review. Summary data for each study included design, data sources, participants, geographic classification system, dates of data collection, clinical management details, and key trends. Extracted data were synthesized descriptively.

### Quality Assessment

The methodological quality of each paper meeting the inclusion criteria was assessed using a tool previously developed for research in breast cancer, based on the Newcastle–Ottawa Scale for assessing quality in non-randomized studies (17, 18). Studies were scored according to the extent that they met each of nine criteria ranging from high risk of bias (score of 0), intermediate risk of bias (score of 1), and low risk of bias (score of 2). Criteria scores were then summed and categorized as “high” (14–18), “moderate” (9–13), or “low” (<9) quality. Study quality appraisal was carried out by two authors, and a third author resolved disagreements between the initial two reviewing authors.

## Results

**Figure 1:**
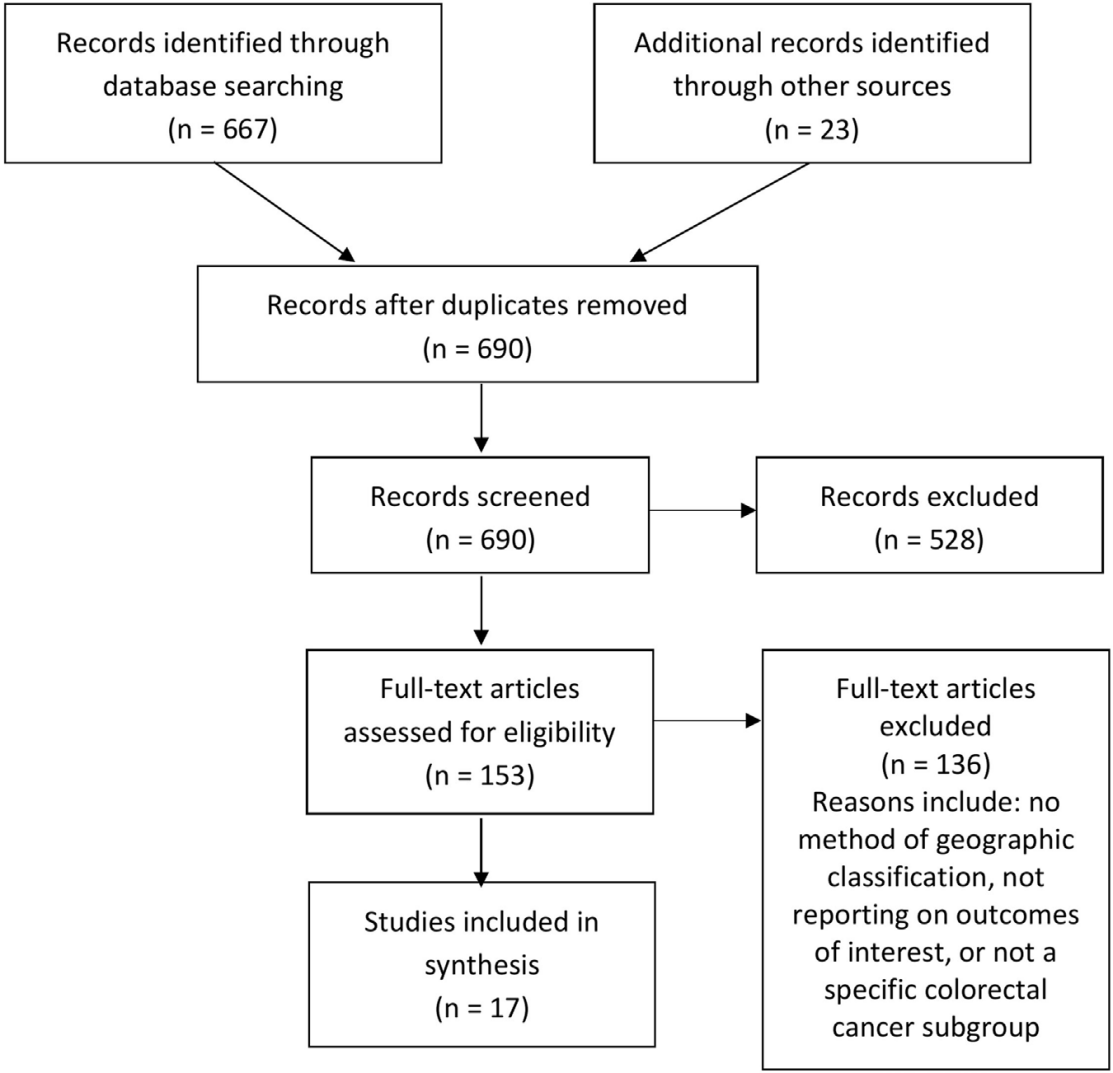
PRISMA flow diagram of included studies.

The search strategy yielded 690 records in total. After removal of duplicates, 681 records were screened by title and abstract. Of those, 153 full-text articles were potentially relevant and assessed for eligibility. Following assessment, 17 studies were included in the final review, comprising 12 peer-reviewed articles and 5 gray literature reports (Figure 1). Because of the diverse nature of the study designs, a quantitative synthesis was not possible and a narrative review of individual studies is provided.

Table 1 presents the key characteristics of all 17 included studies. Eight studies (47%) were of moderate quality, including all five gray literature reports. All other included studies (53%) were high quality indicating they have samples that were representative of the population, variables were collected through secure records, important confounders were controlled for, and statistical methods were appropriate and well presented.

**Table 1:** Characteristics of included studies.

Study	Design and sources	Population	Rural/urban classification	Key findings relating to surgery	Key findings relating to chemotherapy	Key findings relating to radiotherapy	Quality rating
Armstrong et al. (13, 19, 20)	Population-based cross-sectional cohort study New South Wales colorectal cancer care survey	CRC patients (*N* = 3,095 surgery, *N* = 778 chemo, *N* = 238 radiotherapy)	ARIA	Percentage of preoperative investigations by location of residence	Recommended chemotherapy treatment was most likely to be received by patients in “highly accessible” or “accessible” areas (68–69%) compared to “moderately accessible to very remote” patients (43%)	Patients in highly accessible areas had a lower use of radiotherapy (30%) compared to patients in accessible (36%), moderately accessible (35%) and remote/very remote areas (38%)	Moderate
				Highly accessible = 84%, accessible = 14%			
				Moderately accessible = 2%			
				Remote/very remote areas = 1%			

Beckmann et al. (15)	Population-based data linkage study South Australian Cancer Registry, hospital data, radiotherapy databases, hospital-based cancer registries	Residents aged 50 to 79 years with CRC *N* = 4,641	ARIA+ (combined remote/very remote category)	Prevalence ratio of variations in receipt of surgical treatment Inner urban = 1.00 reference outer urban PR = 0.98 Rural PR = 0.99 Remote PR = 1.01	Prevalence ratio of variations in receipt of chemotherapy for stage III CRC Inner urban = 1.00 reference Outer urban PR = 1.03, *p* = 0.63 Rural PR = 0.87, *p* = 0.046 emote PR = 0.86, *p* = 0.14	Prevalence ratio of variations in receipt of radiotherapy	High
						Inner urban PR = 1.00 reference	
						Outer urban PR = 0.87	
						Rural PR = 0.87	
						Remote PR = 1.13	

Beckmann et al. (21)	Population-based data South Australian Cancer Registry, hospital data, and radiotherapy databases	Residents aged 50–79 years diagnosed with CRC who underwent surgical resection (*N* = 3,887; *N* = 3,940 resections)	ARIA (collapsed into metropolitan and non-metropolitan)	No significant differences in risk of post-procedural complications, or risk of readmission were observed in relation to place of residence			High

Chan et al. (22)	Quasi-experimental design using retrospective chart audit, and hospital data from Mt Isa hospital and Townsville Cancer Centre (TCC)	Patients who received chemotherapy at TCC and Mt Isa (*N* = 206). Mt Isa patients received chemotherapy through a tele-oncology model	ASGC classification (Mt Isa = Remote, Townsville = Outer Regional)		There were no significant differences between the Mt Isa and Townsville patients in mean number of treatment cycles, dose intensities, proportions of side effects, and hospital admissions. There were no toxicity-related deaths in either group		High

Clinical Governance Unit (23)	Cross-sectional cohort study National Colorectal Cancer Care Survey	Clinicians treating CRC patients, including surgeons, medical and radiation oncologists (*N* = 2,669)	Patients postcode (capital city, urban, or rural)			Preoperative radiotherapy was more likely to be received by patients in capital city (68%) and urban locations (83%) than rural locations (44%), *p* = 0.004	Moderate

Goldsbury et al. (24)	Retrospective cohort analysis of linked data New South Wales central cancer registry; 45 and Up study; admitted patient data collection; and Medicare claims	Residents participating in the 45 and Up study diagnosed with CRC who had a colonoscopy before diagnosis and surgery after diagnosis (*N* = 407)	Place of residence at diagnosis (metropolitan, other urban, or rural)	Hazard ratio of variation in rectal cancer time to surgery: Rural HR = 0.47; other urban HR = 1.55; metro HR = ref 1.00 Hazard ratio of variation in colon cancer time to surgery: rural HR = 1.21; other urban HR = 1.19; metro HR = ref 1.00 Surgery in a specialist cancer center was more frequent among those living in metro areas (HR = 1.00) compared to other urban areas (HR = 0.27) or rural areas (HR = 0.14)			High

Hall et al. (25)	Retrospective data linkage study West Australian Data Linkage System	Residents with diagnosis of invasive primary CRC *N* = 14,587	ARIA	Patients in remote areas most likely to receive surgery (OR 1.21), compared to very remote (OR 0.70) accessible (OR 1.08), moderately accessible (OR 1.01), and highly accessible (OR 1.00 reference)			High

Henry et al. (26)	Population-based cohort study ECO database (extension of Victorian Cancer Registry, which includes clinical and treatment information)	Residents in the Barwon South Western Region (Victoria) with a cancer diagnosis (*N* = 1,778 for all cancer types)	Distance from Geelong city (km)			Lower radiotherapy utilization was observed for patients living in rural areas compared with those living in Geelong for rectal cancer (32.8 vs 44.7%, *p* = 0.11). Time from diagnosis to radiotherapy was not significantly different for the different geographical regions	Moderate

Hocking et al. (27)	Retrospective cohort study South Australian Clinical Registry for Metastatic Colorectal Cancer	Patients with metastatic CRC (*N* = 2,289)	Postcodes within state capital were “city,” remaining postcodes were “rural”	No significant differences in colorectal surgery (51.5% city vs 55.3% rural, *p* = 0.11), liver surgery (13.7 vs 11.5%, *p* = 0.17), or lung surgery (3.2 vs 2.1%, *p* = 0.10)	Equivalent rates of chemotherapy between metropolitan and rural patients across each line of treatment (56.0 vs 58.3%, respectively, *p* = 0.32). A higher proportion of city patients received combination chemotherapy in the first-line setting (67.4 vs 59.9%; *p* = 0.01). When an oxaliplatin combination was prescribed, oral capecitabine was used more frequently in rural patients (22.9 vs 8.4%; *p* < 0.001)		Moderate

Jorgensen et al. (28)	Linked population-based cohort study New South Wales cancer registry; admitted patients data collection; births, deaths and marriages	Individuals with lymph node-positive colon cancer (*N* = 580) and high-risk rectal cancer (*N* = 498) who underwent surgery following diagnosis	ARIA (remoteness areas); surgeon, and hospital caseload		The majority of the variability in receipt of chemotherapy was attributable to patient characteristics (≈84%), with hospital of surgery accounting for the remaining variability (ICC = 0.16)	Approximately 28% of the total variability in radiotherapy receipt was attributable to hospitals (ICC = 0.28), 2% was attributable to surgeons and the remaining 70% to patient characteristics	High

Morris et al. (29)	Population-based cohort study Pathology records from four West Australian hospitals	Stage III colon cancer patients (*N* = 1,312)	One rural hospital vs three metropolitan hospitals (teaching, private, and district)		Rates of chemotherapy initiation not different between rural hospitals (33.3%) and metropolitan district, private and teaching hospitals (21.1, 47.1, and 31.8%) Rates of chemotherapy completion not different between metropolitan district hospitals and rural hospitals (48.4 vs 45.2%, respectively), but higher in metropolitan private and teaching hospitals (73.3 and 69.3%)		High

Pathmanathan et al. (30)	Retrospective clinical chart audit Hospital records (Townsville Cancer Center)	Patients from Townsville or North West Queensland districts aged > 18 years diagnosed with colorectal cancer (*N* = 51)	RRMA 3 ≥3 classified as rural, RRMA 2 (Townsville) classified as urban		A similar number of patients received XELOX as a second-line treatment in urban (*n* = 10; 40%) and rural (*n* = 8; 31%) areas with a similar number of cycles (urban *n* = 49; 31% vs rural *n* = 37; 24%). No differences in dose intensities were apparent		Moderate

Queensland Government (31)	Retrospective population-based audit Queensland Oncology Repository	Queensland patients diagnosed with colon (*N* = 1,537) and rectal (*N* = 656) cancer who had a major resection	ASGC	Colon cancer % days from diagnosis to surgery ≤30 Major city = 77%; inner regional = 74%; outer regional = 70%; remote = 70% Rectal cancer % days from diagnosis to surgery ≤30 Major city = 44%; inner regional = 34%; outer regional = 35%; remote = 21%			Moderate

Singla et al. (32)	Retrospective cohort study South Australian Clinical Registry for Metastatic Colorectal Cancer	SA patients with metastatic CRC (*N* = 2,001)	ASGC	No significant differences between major city, inner regional, outer regional, and remote patients in rates of lung surgery (1.8, 3.8, 1.1, and 1.0% respectively; *p* = 0.104) or liver surgery (13.0, 13.2, 13.6, and 9.4%; *p* = 0.753)			High

Young et al. (33)	Prospective audit New South Wales Central Cancer Registry	NSW patients newly diagnosed with CRC (*N* = 3,095)	Hospital location (metropolitan or rural)		Patients offered recommended adjuvant chemotherapy for colon cancer were more likely to be treated in a metropolitan hospital than rural hospital (OR = 1.00 vs OR = 0.56, *p* = 0.04)		High

*ARIA = Accessibility/Remoteness Index of Australia; ASGC = Australian Standard Geographical Classification; CRC = colorectal cancer; HR = hazard ratio; OR = odds ratio; PR = prevalence ratio; RRMA = Rural, Remote, Metropolitan Area*.

### Main Findings

#### Surgery

Eight studies reported on surgical management. Three studies reported no significant variation in rates of surgical treatment according to place of residence, for all stage cancers (15) or metastatic disease (27, 32). Five studies reported geographical variations in aspects of surgical management, although methodologies varied greatly. A retrospective data linkage study found that patients residing in remote areas of Western Australia were more likely to receive surgery compared to patients residing in any other area (25). However, when location of hospital was considered, patients were more likely to receive surgery when their first admission was to a metropolitan facility rather than a rural hospital (25, 28). A Queensland audit report suggested that it was more likely for patients residing in metropolitan areas to have surgery less than 30 days after diagnosis, compared to patients in outer regional and remote areas (31). A retrospective cohort analysis from New South Wales reported that among rectal cancer patients there was a longer delay until surgery for individuals living in rural areas, but this was not the case for colon cancer patients (24). A clinicians report from NSW examined preoperative investigations, and reported that patients residing in highly accessible areas were significantly more likely to have had recommended tests such as colonoscopy, sigmoidoscopy, and scans for distant metastases compared to those living in less accessible areas (13).

#### Chemotherapy

Eight studies examined chemotherapy management. Three studies indicated that chemotherapy for stage III colon cancer was less likely to be received by patients residing in rural and remote areas than metropolitan areas (15, 19, 33). One study found equivalent rates of chemotherapy management between patients residing in metropolitan and rural areas across each line of treatment, although higher proportions of metropolitan patients received combination chemotherapy in the first-line, and rural patients had increased use of the oral prodrug capecitabine as first-line treatment compared to metropolitan patients (27). In contrast, two Queensland studies reported a similar number of chemotherapy cycles, regimen types, and dose-intensities used in both rural and urban areas (22, 30). Two retrospective cohort studies reported no geographical variation in receipt of chemotherapy (28, 29).

#### Radiotherapy

Five studies examined radiotherapy management in rectal cancer patients, with four reporting geographical disparities in radiotherapy use. Specifically, one retrospective cohort study reported lower utilization of radiotherapy in patients residing in rural Victoria that was not explained by age; a pattern that was more prominent in men than women (26). One cohort study conducted in New South Wales reported variability in receipt of radiotherapy as being attributable to location of hospital rather than place of residence, with radiotherapy less likely to occur in rural hospitals (28). In contrast, a New South Wales study of clinician reports suggested that patients residing in highly accessible areas had lower use of radiotherapy than non-metropolitan areas; however, this study had small numbers of rectal cancer patients in moderately accessible and remote areas (20). One national cross-sectional study reported that patients residing in rural areas were less likely to receive preoperative radiotherapy for high-risk rectal cancer although it is unclear whether these patterns are indicative of patient characteristics, physician recommendations, or health service accessibility (23). Only one retrospective data-linkage study found no differences in radiotherapy for stage II and stage III rectal cancer according to location of residence in South Australia (15).

## Discussion

The current review found inconsistent evidence relating to geographical disparities in clinical management of CRC in Australia. While some studies showed no differences in treatment by location of residence, other studies reported that patients with CRC in non-metropolitan areas of Australia are less likely to receive optimal care. This was particularly true for studies where the outcome was radiotherapy utilization. The evidence gathered in this review highlights key issues with consistency in current data collection and reporting regarding CRC treatments and clinical management. In particular, the review highlights the importance of recording location of treatment as well as location of residence; gaps in data collection at a population-level; and large variability in the methodologies used to investigate and report on geographical disparity. There is significant capacity for future research to focus on these critical issues, as well as aspects missing from the current literature such as treatment completion rates and reasons for non-receipt of adjuvant therapies.

Population level data collected in cancer registries internationally provides information on all cancers occurring in a certain population (34). The type of data that cancer registries can provide is varied, and while most provide information on incidence and mortality, currently many registries, including those in Australia, do not routinely collect data on stage at diagnosis or treatment details (35). Hospital registries and other sources of clinical data may have this information available, but it is generally limited to a single institution or health service area. In clinical practice, factors such as position of the tumor, lymph node invasion, involved margins, as well as individual factors such as age, and comorbidities influence CRC treatment decisions (4, 36), yet, detailed clinical information is not available at the population level. This significantly hampers research efforts attempting to understand disparities in CRC management. The findings of the current review emphasize a need for more comprehensive data collection, particularly, in this era of electronic data. This might involve the use of standardized reporting database software such as electronic health records, whereby variables such as patients’ residence, hospital location, treatment type, tumor stage, and comorbidities are recorded and able to be linked to cancer registry data. As the use of electronic health records and capacity for data-linkage expands in Australia, and worldwide, these provide a useful avenue for the collection of clinical management data for CRC patients, with the ability to share information across networks. The digital collection of cancer management data at the population level could result in complex databases to be used for a multitude of previously unanswered research questions (37).

Several studies reported that clinical management for CRC was less optimal in non-metropolitan areas of Australia. For instance, some studies reported that when non-metropolitan patients underwent surgery for CRC, it was more likely to be delayed and less likely to be preceded by preoperative investigations, radiotherapy for rectal cancer patients was underutilized in non-metropolitan areas, and non-metropolitan patients with stage III colon cancer were less likely to receive recommended chemotherapy treatment. Despite potential differences in the provision of chemotherapy across geographic locations, chemotherapy regimens were generally found to be similar across metropolitan and non-metropolitan areas (22, 30). However, one study indicated that oral chemotherapy was more likely to be used for rural patients with metastatic disease (27). The advantages of oral regimens over chemotherapy by infusion include convenience, flexibility in location of administration, and reduced toxicity-related hospitalization, which may be of benefit to patients in non-metropolitan areas (38–40). For these reasons, the use of innovative chemotherapy regimens is a promising solution for overcoming the barrier of distance for optimal CRC treatment.

Previous studies in Canada, the United States, and the United Kingdom have also reported less optimal treatment provided to non-metropolitan CRC patients (41–43), although again evidence is mixed (41). A number of international studies have reported that geographic variability in the treatment of CRC may be reflective of the different population compositions in regional areas, as well as hospital volume and service availability. For instance, older patients, black patients, and patients of lower socioeconomic status (SES) were less likely to receive recommended treatments and more likely to have poorer outcomes (43–47). As rural Australia is also characterized by populations with lower education, lower SES, more advanced age, and higher rates of Indigenous populations (12, 48), these sociodemographic factors may explain many of the treatment differences reported in the current review. If SES, ethnicity, and demographic characteristics are explanations for geographical disparity in cancer treatment resulting in poorer cancer outcomes, there is an issue of inequality that needs to be urgently addressed.

Studies that reported data comparing regional and metropolitan facilities, rather than basing geographic location on patient’s home address, generally reported less optimal clinical management in non-metropolitan areas. This may suggest that there are important differences in the quality of treatment for patients receiving treatment in regional areas. If disparities in clinical management of CRC are explained to some extent by accessibility to appropriate services, then changes to health service delivery may be an appropriate solution (11, 49). To date, increased use of telehealth, the development of approved oral chemotherapy regimens, and the requirement for overseas trained health workers to work in rural and regional areas are strategies implemented to help overcome the challenge of distance (38, 50–52). The results of this review suggest that planning for health service delivery must continue to adapt and focus on overcoming barriers due to distance. In particular, high priority areas are the recruitment and retention of specialist staff in non-metropolitan areas, reduction of wait times, and tailoring services to individuals of low SES, increased age, multiple comorbidities, and indigenous populations.

Based on evidence presented above, it appears that clinical management of CRC in non-metropolitan Australia may be less than optimal; however, this may be due to a range of patient and provider factors that correspond to geographic location. As evidence in this area is limited, one major contribution of the current review is to motivate future research. Future studies will need to collect more detailed data on clinical indicators, health professionals decision-making, and patient preferences to shed light on potential variations in CRC treatment. To provide optimal treatment equitably to patients with CRC, a better understanding of the underlying causes of geographical variations in treatment is required. Digital data collection tools provide an opportunity to address this. Further investigation into the relative contribution of patient, provider, and health system factors to geographical disparities in clinical management is essential. Subsequently, interventions designed to improve the quality of care can then be directed at those patients most likely to benefit from them, such as the provision of extra care from nurse navigators by assessing and monitoring “at risk” patients. Additionally, health services can use this information to adapt appropriately to suit the needs of regional populations, for instance, through the extended use of tele-oncology models of care, oral chemotherapy regimens, or the development of outreach radiotherapy facilities to improve quality of care for those living outside city centers. Not only is this of relevance in Australia but also to countries with similar geographic distributions and population characteristics such as the US, Canada, and the UK.

### Limitations

This review is limited by the small number of studies and the use of inconsistent methodology across studies. The variation in population samples, and use of different geographical classifications made direct comparisons between studies difficult. Above all, research identifying geographical disparities in cancer outcomes is hindered by the lack of accurate patient and treatment data at a population level. The use of digital systems to collect and record clinical management information would enhance understanding of variations in cancer outcomes and inform policy and clinical guidelines. The reviewed studies emphasize the need for better data collection and reporting, and highlight the need for the use of data linkage to gather comprehensive clinical management data.

### Conclusion

The present review provides specific information on clinical management differences across geographic regions in Australia. This synthesis of existing literature highlights significant issues both for data collection and reporting at the population level, an issue of relevance worldwide. Improvements in cancer outcomes in regional areas will require enhanced capacity to accurately track, and respond to, geographical disparities. Through the use of electronic health records, data linkage and future research, it is important to investigate differences in clinical management of CRC across geographical locations, and in particular, the patient, professional, or health service factors, which contribute to these disparities. Causes for disparities in treatment are found not only in the individual patients and their social environments but also in the location and quality of the health-care system. It is recommended that population-level data regarding clinical management of CRC is routinely collected to improve health outcomes and inform future guidelines and policy.

## Author Contributions

All authors have made substantial contributions to acquisition, analysis, and interpretation of data, and drafting the manuscript. JD, SC, SM, and JA provided substantial contribution to conception of the work, and revised the work for important intellectual content. FC-W, AR, and MI carried out searches, screening, and extraction. Study quality appraisal was carried out by AR and FC-W and moderated by MI. BG and MH contributed to conception of work, edited, and critically revised the manuscript. Each author has participated sufficiently in the work and takes responsibility for appropriate portions of the content. All authors have read and have given final approval of the version to be published.

## Conflict of Interest Statement

The authors declare that the preparation of this article was conducted in the absence of any commercial or financial relationships that could be construed as a potential conflict of interest. The reviewers DG and RK and the handling Editor declared their shared affiliation.

## References

[1] Australian Institute of Health and Welfare. Cancer in Australia 2017. Canberra: AIHW (2017).

[2] Ferlay J Soerjomataram I Ervik M Dikshit R Eser S Mathers C GLOBOCAN 2012: Cancer Incidence and Mortality Worldwide. Lyon, France: International Agency for Research on Cancer (2014) [cited 2015 Jan 16]. Available from: http://globocan.iarc.fr

[3] ACIM. Australian Cancer Incidence and Mortality (ACIM) Books: Colorectal Cancer. Canberra: AIHW (2016).

[4] National Health and Medical Research Council. Guidelines for the Prevention, Early Detection and Management of Colorectal Cancer. Sydney: The Cancer Council Australia and Australian Cancer Network (2005).

[5] Department of Health and Human Services. Optimal Care Pathway for People with Colorectal Cancer. Melbourne, Australia: Victorian Government (2015).

[6] Valery PC Coory M Stirling J Green AC Cancer diagnosis, treatment, and survival in Indigenous and non-Indigenous Australians: a matched cohort study. Lancet (2006) 367(9525):1842–8. 10.1016/S0140-6736(06)68806-5 16753487

[7] Cunningham J Rumbold AR Zhang X Condon JR. Incidence, aetiology, and outcomes of cancer in Indigenous peoples in Australia. Lancet Oncol (2008) 9(6):585–95. 10.1016/S1470-2045(08)70150-5 18510990

[8] Ireland M March S Crawford-Williams F Cassimatis M Aitken JF Hyde MK A systematic review of geographical differences in management and outcomes for colorectal cancer in Australia. BMC Cancer (2017) 17:95. 10.1186/s12885-017-3067-1 28152983 PMC5290650

[9] Australian Institute of Health and Welfare. Radiotherapy in Australia 2015-16. Canberra: AIHW (2017).

[10] Morgan GW Barton M Atkinson C Millar J Kumar Gogna N Yeoh E ‘GAP’ in radiotherapy services in Australia and New Zealand in 2009. J Med Imaging Radiat Oncol (2010) 54(3):287–97. 10.1111/j.1754-9485.2010.02172.x 20598017

[11] Underhill C Bartel R Goldstein D Snodgrass H Begbie S Yates P Mapping oncology services in regional and rural Australia. Aust J Rural Health (2009) 17(6):321–9. 10.1111/j.1440-1584.2009.01106.x 19930199

[12] Australian Bureau Statistics. Australian Social Trends. Canberra: ABS (2013).

[13] Armstrong K O’Connell D Leong D Spigelman A Armstrong BK The New South Wales Colorectal Cancer Care Survey 2000 – Part 1 Surgical Management. Kings Cross, NSW: The Cancer Council NSW (2004).

[14] Wilson A Marlow NE Maddern GJ Barraclough B Collier NA Dickinson IC Radical prostatectomy: a systematic review of the impact of hospital and surgeon volume on patient outcome. ANZ J Surg (2010) 80(1–2):24–9. 10.1111/j.1445-2197.2009.05172.x 20575876

[15] Beckmann KR Bennett A Young GP Roder DM Treatment patterns among colorectal cancer patients in South Australia: a demonstration of the utility of population-based data linkage. J Eval Clin Pract (2014) 20(4):467–77. 10.1111/jep.12183 24851796

[16] Liberati A Altman DG Tetzlaff J Mulrow C Gøtzsche PC Ioannidis JPA The PRISMA statement for reporting systematic reviews and meta-analyses of studies that evaluate healthcare interventions: explanation and elaboration. BMJ (2009) 339:b2700. 10.1136/bmj.b2700 19622552 PMC2714672

[17] Youl PH Dasgupta P Youlden D Aitken JF Garvey G Zorbas H A systematic review of inequalities in psychosocial outcomes for women with breast cancer according to residential location and Indigenous status in Australia. Psychooncology (2016) 25(10):1157–67. 10.1002/pon.4124 26989048

[18] Wells G Shea B O’connell D Peterson J Welch V Losos M The Newcastle-Ottawa Scale (NOS) for Assessing the Quality of Nonrandomised Studies in Meta-Analyses. Ottawa: Ottawa Health Research Institute (1999) [cited 2016 May 11]. Available from: http://www.ohri.ca/programs/clinical_epidemiology/oxford.asp

[19] Armstrong K O’Connell D Leong D Spigelman A Armstrong BK The New South Wales Colorectal Cancer Care Survey 2000 – Part 2 Chemotherapy Management. Kings Cross, NSW: The Cancer Council NSW (2005).

[20] Armstrong K O’Connell D Leong D Spigelman A Armstrong BK The New South Wales Colorectal Cancer Care Survey 2000 – Part 3 Radiotherapy Management. Kings Cross, NSW: The Cancer Council NSW (2007).

[21] Beckmann KR Bennett A Young GP Cole SR Joshi R Adams J Sociodemographic disparities in survival from colorectal cancer in South Australia: a population-wide data linkage study. BMC Health Serv Res (2016) 16:24 10.1186/s12913-016-1263-3 26792195 PMC4721049

[22] Chan BA Larkins SL Evans R Watt K Sabesan S. Do teleoncology models of care enable safe delivery of chemotherapy in rural towns? Med J Aust (2015) 203(10):406–6.e6. 10.5694/mja15.00190 26561905

[23] Clinical Governance Unit. The National Colorectal Cancer Care Survey: Australian Clinical Practice in 2000. Melbourne, Australia: National Cancer Control Initiative (2002).

[24] Goldsbury D Harris MF Pascoe S Olver I Barton M Spigelman A Socio-demographic and other patient characteristics associated with time between colonoscopy and surgery, and choice of treatment centre for colorectal cancer: a retrospective cohort study. BMJ Open (2012) 2(3):e001070. 10.1136/bmjopen-2012-001070 22637491 PMC3367154

[25] Hall SE Holman CD Platell C Sheiner H Threlfall T Semmens J Colorectal cancer surgical care and survival: do private health insurance, socioeconomic and locational status make a difference? ANZ J Surg (2005) 75(11):929–35. 10.1111/j.1445-2197.2005.03583.x 16336380

[26] Henry MJ Jones P Morrissy K Matheson LM Pitson G Healy P Radiotherapy in the Barwon South Western Region: a rural perspective. J Med Imaging Radiat Oncol (2014) 58(5):612–7. 10.1111/1754-9485.12208 25091019

[27] Hocking C Broadbridge VT Karapetis C Beeke C Padbury R Maddern GJ Equivalence of outcomes for rural and metropolitan patients with metastatic colorectal cancer in South Australia. Med J Aust (2014) 201(8):462–6. 10.5694/mja14.00046 25332033

[28] Jorgensen ML Young JM Dobbins TA Solomon MJ. Does patient age still affect receipt of adjuvant therapy for colorectal cancer in New South Wales, Australia? J Geriatr Oncol (2014) 5(3):323–30. 10.1016/j.jgo.2014.02.007 24656735

[29] Morris M Platell C Fritschi L Iacopetta B. Failure to complete adjuvant chemotherapy is associated with adverse survival in stage III colon cancer patients. Br J Cancer (2007) 96:701–7. 10.1038/sj.bjc.6603627 17299387 PMC2360063

[30] Pathmanathan S Burgher B Sabesan S. Is intensive chemotherapy safe for rural cancer patients? Int Med J (2013) 43(6):643–9. 10.1111/imj.12083 23347337

[31] Queensland Government. Queensland Colorectal Cancer Audit 2016. Brisbane: Queensland Cancer Control Analysis Team (2016).

[32] Singla A Broadbridge V Mittinty M Beeke C Maddern GJ. Rural populations have equal surgical and survival outcomes in metastatic colorectal cancer. Aust J Rural Health (2014) 22(5):249–56. 10.1111/ajr.12133 25303417

[33] Young JM Leong DC Armstrong K O’Connell D Armstrong BK Spigelman AD Concordance with national guidelines for colorectal cancer care in New South Wales: a population-based patterns of care study. Med J Aust (2007) 186:292–5. 17371209 10.5694/j.1326-5377.2007.tb00903.x

[34] Silva I Reality TV. In: Creber G, editor. The Television Genre Book. London: British Film Institute (1999). p. 134–7.

[35] Forsea A-M Cancer registries in Europe – going forward is the only option. Ecancermedicalscience (2016) 10:641 10.3332/ecancer.2016.641 27350787 PMC4898937

[36] Labianca R Nordlinger B Beretta GD Mosconi S Mandalà M Cervantes A Early colon cancer: ESMO clinical practice guidelines for diagnosis, treatment and follow-up. Ann Oncol (2013) 24(Suppl_6):vi64–72. 10.1093/annonc/mdt354 24078664

[37] Fleming M Kirby B Penny KI. Record linkage in Scotland and its applications to health research. J Clin Nurs (2012) 21(19–20):2711–21. 10.1111/j.1365-2702.2011.04021.x 22985317

[38] Sabesan S Larkins S Evans R Varma S Andrews A Beuttner P Telemedicine for rural cancer care in North Queensland: bringing cancer care home. Aust J Rural Health (2012) 20(5):259–64. 10.1111/j.1440-1584.2012.01299.x 22998200

[39] Malet-Martino M Martino R. Clinical studies of three oral prodrugs of 5-fluorouracil (capecitabine, UFT, S-1): a review. Oncologist (2002) 7(4):288–323. 10.1634/theoncologist.7-4-288 12185293

[40] Bhattacharyya GS Oral systemic therapy: not all “win-win”. Indian J Med Paediatric Oncol (2010) 31(1):1–3. 10.4103/0971-5851.68844 PMC2941596 20931012

[41] Campbell NC Elliott AM Sharp L Ritchie LD Cassidy J Little J. Impact of deprivation and rural residence on treatment of colorectal and lung cancer. Br J Cancer (2002) 87(6):585–90. 10.1038/sj.bjc.6600515 12237766 PMC2364239

[42] Eldin NS Yasui Y Scarfe A Winget M Adherence to treatment guidelines in stage II/III rectal cancer in Alberta, Canada. Clin Oncol (2012) 24(1):e9–17. 10.1016/j.clon.2011.07.005 21802914

[43] Sankaranarayanan J Watanabe-Galloway S Sun J Qiu F Boilesen EC Thorson AG. Age and rural residence effects on accessing colorectal cancer treatments: a registry study. Am J Manag Care (2010) 16(4):265–73. 20394462

[44] Ayanian JZ Zaslavsky AM Fuchs CS Guadagnoli E Creech CM Cress RD Use of adjuvant chemotherapy and radiation therapy for colorectal cancer in a population-based cohort. J Clin Oncol (2003) 21(7):1293–300. 10.1200/jco.2003.06.178 12663717

[45] Hines RB Markossian TW. Differences in late-stage diagnosis, treatment, and colorectal cancer-related death between rural and urban African Americans and whites in Georgia. J Rural Health (2012) 28(3):296–305. 10.1111/j.1748-0361.2011.00390.x 22757954

[46] Le H Ziogas A Lipkin SM Zell JA Effects of socioeconomic status and treatment disparities in colorectal cancer survival. Cancer Epidemiol Prev Biomarkers (2008) 17(8):1950–62. 10.1158/1055-9965.EPI-07-2774 18708384

[47] Aarts MJ Lemmens VE Louwman MW Kunst AE Coebergh JW. Socioeconomic status and changing inequalities in colorectal cancer? A review of the associations with risk, treatment and outcome. Eur J Cancer (2010) 46(15):2681–95. 10.1016/j.ejca.2010.04.026 20570136

[48] Baxter J Gray M Hayes A Families in Regional, Rural, and Remote. Australia, Melbourne: AIFS (2011).

[49] Amgen Australia Pty Ltd. Access to Cancer Treatment in Non-Metropolitan Areas of Australia. Barton, ACT: Deloitte Access Economics (2011) [cited 2016 Aug 4]. Available from: https://www2.deloitte.com/au/en/pages/economics/articles/access-cancer-treatment-non-metropolitan-australia.html

[50] Department of Health. Review of Australian Government Health Workforce Programs. International Recruitment, Support and Regulation. Canberra, Australia: Department of Health (2013) [updated 2013 May 24; cited 2017 Jan 20]. Available from: http://www.health.gov.au/internet/publications/publishing.nsf/Content/work-review-australian-government-health-workforce-programs-toc~chapter-6-managing-supply-health-workers-meet-community-needs~chapter-6-international-recruitment-support-regulation

[51] Chapman A Shakespeare T Turner M Improving access to radiotherapy for regional cancer patients – the National Radiotherapy Single Machine Unit Trial. Cancer Forum (2007) 31(2):74–7.

[52] Hoff PM Ansari R Batist G Cox J Kocha W Kuperminc M Comparison of oral capecitabine versus intravenous fluorouracil plus leucovorin as first-line treatment in 605 patients with metastatic colorectal cancer: results of a randomized phase III study. J Clin Oncol (2001) 19(8):2282–92. 10.1200/JCO.2001.19.8.2282 11304782

